# Impact of DOTS expansion on tuberculosis related outcomes and costs in Haiti

**DOI:** 10.1186/1471-2458-6-209

**Published:** 2006-08-15

**Authors:** Vary Jacquet, Willy Morose, Kevin Schwartzman, Olivia Oxlade, Graham Barr, Franque Grimard, Dick Menzies

**Affiliations:** 1 National tuberculosis control programme, Port-au-Prince, Haiti; 2 Respiratory Epidemiology Unit, Montreal Chest Institute, McGill University, Montreal, Canada; 3 Departments of Medicine and Epidemiology, Columbia University Medical Center, New York, NY, USA; 4 Department of Economics, McGill University, Montreal, Canada

## Abstract

**Background:**

Implementation of the World Health Organization's DOTS strategy (Directly Observed Treatment Short-course therapy) can result in significant reduction in tuberculosis incidence. We estimated potential costs and benefits of DOTS expansion in Haiti from the government, and societal perspectives.

**Methods:**

Using decision analysis incorporating multiple Markov processes (Markov modelling), we compared expected tuberculosis morbidity, mortality and costs in Haiti with DOTS expansion to reach all of the country, and achieve WHO benchmarks, or if the current situation did not change. Probabilities of tuberculosis related outcomes were derived from the published literature. Government health expenditures, patient and family costs were measured in direct surveys in Haiti and expressed in 2003 US$.

**Results:**

Starting in 2003, DOTS expansion in Haiti is anticipated to cost $4.2 million and result in 63,080 fewer tuberculosis cases, 53,120 fewer tuberculosis deaths, and net societal savings of $131 million, over 20 years. Current government spending for tuberculosis is high, relative to the per capita income, and would be only slightly lower with DOTS. Societal savings would begin within 4 years, and would be substantial in all scenarios considered, including higher HIV seroprevalence or drug resistance, unchanged incidence following DOTS expansion, or doubling of initial and ongoing costs for DOTS expansion.

**Conclusion:**

A modest investment for DOTS expansion in Haiti would provide considerable humanitarian benefit by reducing tuberculosis-related morbidity, mortality and costs for patients and their families. These benefits, together with projected minimal Haitian government savings, argue strongly for donor support for DOTS expansion.

## Background

Between 1997 and 2002 the incidence of active TB increased in most low and middle income countries [1]. This occurred despite the availability of adequate tools for diagnosis and treatment, and an effective TB control strategy – which has been labelled DOTS. This strategy, originally developed in sub-Saharan Africa, is being promoted by the World Health Organisation (WHO) [2] because it is feasible in high-burden settings [3], cost-effective even in low income countries [4], and can result in substantial reduction in TB incidence [5].

However, between $290 – $500 million (US) is required annually for the additional training, equipment and infrastructure needed to implement and maintain DOTS in all low and middle income countries [6,7]. As a result DOTS expansion has lagged considerably behind WHO targets [8].

In the America's, Haiti is the poorest country [9], with the highest incidence of smear positive pulmonary TB (138/100,000 in 2002) [8] and HIV seroprevalence (4.5% in 2002) [10]. In 2002 the WHO estimated that only 37% of the population had access to DOTS programmes [11], only 49% of all smear positive cases were diagnosed, and only 71% of those diagnosed were successfully treated [8]. We have compared projected TB related outcomes and costs if the current control programme is maintained (status quo), or if DOTS is expanded to reach WHO benchmarks, for 100% of the population in Haiti.

## Methods

### General description of model and the two strategies compared

We developed a decision analysis model, incorporating multiple Markov processes, (Markov modelling) using Tree-Age Pro Release 6.0, (Tree-age Inc., Williamstown, MA). This model calculated the probability of TB-related events expected to result over 20 years, starting in 2003, from two alternate strategies for TB control. These probabilities were calculated for a hypothetical fixed cohort with the age structure, socio-demographic and health characteristics and size of the population of Haiti in 2002. The information on the population of Haiti, summarized in Table 1, was taken from publicly accessible information provided by international agencies including the World Bank, WHO, and the United Nations Program on AIDS (UNAIDS) [8-10].

The strategies compared were to continue the present national TB control programme (status quo), or DOTS expansion. With the status quo strategy there would be no change over the 20 year time frame of the analysis, from January 2003 levels of: DOTS coverage [11], case finding [8], treatment outcomes [8], incidence of smear positive TB [8], prevalence of initial TB drug resistance [12,13], and HIV seroprevalence [10]. Since incidence of disease did not change with the status quo, risk of TB infection [14], and LTBI prevalence also remained unchanged throughout the period of analysis. Current levels of DOTS coverage have been achieved with assistance from foreign donors – hence we implicitly accounted for current foreign assistance. However, we did not explicitly add in these expenditures, since they would have been added to both strategies, making both more expensive, but without changing the differences between them.

With DOTS expansion, we assumed that DOTS would be expanded from the level of coverage in January 2003, (Year 1) [11], to reach 100% of government health facilities by the end of Year 3. Case detection would increase from current WHO estimated levels [8] to 70%, and treatment success (cure and treatment completion) to 85% – the WHO targets [3], although treatment failure and TB mortality would not change [8]. Rates of initial drug resistance [12,13] and HIV prevalence [10] would not change with DOTS over the full 20 years. DOTS implementation would halve pre-diagnostic health system visits [15,16], and health system delays [17-19], although patient delays would not change. Hospitalisation duration would decrease by two-thirds [15,16]. The number of patients investigated for each new case of TB diagnosed would increase from 4 to 16 [20]. Retreatment failures (multi-drug resistant TB) would be treated with second line drugs obtained from the Green Light Committee [21] with 48% cure, and 12% mortality [22]. Most importantly DOTS expansion was assumed to result in a 6% annual decline in incidence, as described in Peru following national DOTS implementation [5]. This would produce corresponding reductions in the risk, and prevalence of TB infection [14].

### Health states and transitional probabilities (see Figure 1)

At the start of Year 1 (assumed to be 2003), cohort members were considered to be in one of five TB-related states, and one of three HIV-related states. The TB-related states were: 1) no tuberculosis infection; 2) recent latent tuberculosis infection (LTBI) – acquired within 2 years; and, 3) long-standing LTBI – acquired more than 2 years ago; 4) active tuberculosis; and, 5) treated, or spontaneously resolved active TB. States 2–5 were further sub-classified into 3 groups: drug-sensitive, single-drug resistant, or multi-drug resistant TB – with likelihood based on surveys of drug resistance in Haitian populations [12,13]. The proportion with recent or long-standing LTBI was calculated based on the age structure of the population [23], and incidence of smear-positive active disease [8], using the Styblo formula [14].

Key pathogenetic model assumptions regarding reactivation and cure rates for HIV-negative and HIV-positive individuals, summarized in Table 2, were based on published cohort studies and randomized trials. In HIV negative persons, active TB would develop in the first two years after new TB infection in 5% of the population [24]. In other studies the risk of disease in the first two years after new TB infection ranged from 4% [25] to 10% [26]. Given the 80% protective effect afforded by previous latent TB infection in HIV uninfected persons [27], re-infection would be followed by a 1% risk of active TB in the first two years. In latently infected persons risk would be 0.1% annually after the first two years – based on prospective follow-up of two tuberculin positive cohorts – of young military recruits [28], and Vietnamese refugees [29].

The probability of transfer out and default varied by strategy. We assumed that the treatment outcome of "transferred-out" was equivalent to default [30]. For individuals who defaulted from therapy we assumed an overall cure rate of 62%, calculated from the proportion of defaulters after different lengths of therapy [31], and cure rates in randomised trials of regimens of 3 or 4 months duration [32-34].

The HIV-related states were: 1) no HIV infection; 2) early HIV, defined as having no clinical manifestations; or, 3) late HIV infection – defined as clinical AIDS. HIV-related survival and annual rates of transition from early to late HIV states were based on a Ugandan cohort [35]. Probability of acquiring HIV infection was the same in both strategies. Annual risk of HIV infection was estimated to be 0.49% – calculated from the general population prevalence [10] divided by the average years of survival in a low-income setting [35] (and thus assumed to be uniform for the entire population). We assumed no consequence of HIV re-infection.

The same model was used for HIV infected or uninfected. The proportion of HIV infected persons entering the model was 0.045 – corresponding to the estimated seroprevalence in Haiti in 2002 (10). Every year, 0.5% of the uninfected population could acquire new HIV infection, thus changing from an HIV uninfected to an HIV infected state. The major difference in the model for HIV infected and uninfected was in regard to the probability of development of active TB following TB infection and mortality – without TB, or during and after treatment for active TB. The risk of active TB in persons with LTBI, and HIV infection has been studied in a number of settings. We could find only one study that categorized cohort members into the states of new or old TB infection and early or late HIV [36]. This was used for the base case estimate of 3.4% annual risk of active TB disease in persons with long-standing LTBI and HIV infection.

We could not find published estimates of risk of disease following new TB infection in HIV infected individuals. Therefore we extrapolated from HIV negative persons, in whom the risk of development of active TB following new LTBI infection is 20–50 times higher than with long-standing LTBI [24-26]. Given the 3.4% annual risk of active TB in early HIV infection and long-standing LTBI [36], we assumed the risk of TB disease would be 10 times higher, or 34% per year during the first two years following new TB infection.

Response to treatment of TB disease was not affected by HIV infection. HIV-infected individuals with smear positive active TB were assumed to have no chance of spontaneous cure – hence 100% mortality without treatment. If treated, TB mortality would be 2.25 times higher during treatment [37-39], and 2.2 times higher after treatment [3,40,41]. Mortality from active TB disease with MDR strains would be 100% [42,43].

### Model calculations

Beginning in 2003 (Year 1), the model determined the proportion of the cohort developing smear positive active TB, dying from TB, or dying from other causes in each year for 20 successive years. The risk of death from other causes was derived from the age distribution and HIV sero-prevalence of the cohort, and country-specific life tables published by WHO [44]. The probability of TB related outcomes depended on cohort members' TB and HIV related states (infected or not, and new or old), as detailed above, and summarized in Table 2. Clinical outcomes also varied according to whether each TB related state was diagnosed and treated.

Cohort members who survived to the end of each year in the model, entered the following year of the simulation. Health states at the beginning of each year depended on the events during the preceding year. As a (simplified) example, some members of the cohort entered Year 1 in the health state of no TB infection. If they survived Year 1 without acquiring TB or HIV infection they entered Year 2 in the same state. However if they acquired new TB infection during Year 1, they entered Year 2 in the "new TB infection" state. During Year 2, they could develop active TB (with probability as shown in Table 2), die from other causes, or remain with new TB infection (entering Year 3 with this health state). If they developed active TB they could be diagnosed and treated, or remain undiagnosed. Likelihood of diagnosis, and treatment outcomes varied according to the strategy being analyzed (see Table 1), and underlying drug resistance. The probability of having pan- sensitive, single drug resistant, or multi-drug resistant underlying strains, was determined from two surveys of initial drug resistance in Haitian TB patients [12,13]. If undiagnosed they could die of TB (probability in Table 2) or other causes (from Life Tables), or survive and enter Year 3 with undiagnosed TB disease.

The model generated a single value for the cumulative proportion of the cohort that developed active TB, died of TB, or died of other causes throughout the 20 years for each strategy. This was then multiplied by the size of the population to generate expected total number of persons developing each outcome over the period of analysis.

### Costs

Costs were estimated from societal, and governmental perspectives [45]. Costs modelled from the government perspective included TB-related health care costs for governments and health care providers, plus costs for DOTS implementation, maintenance, and drug costs. TB related costs from the societal perspective included government costs, plus patients' and families' out-of-pocket expenditures, lost wages (including time caring for ill family members), plus productivity losses resulting from disability and death [45]. All costs were expressed in 2003 US dollars, and all future expenditures and outcomes were discounted 3% annually [46].

A questionnaire regarding the household impact of fatal adult illness [47] was adapted, pre-tested in Montreal, and translated into French and Creole – to measure out-of-pocket costs and lost income for patients and families for pre-diagnostic, hospitalisation, treatment and follow-up visits. This was administered by trained interviewers to 84 consenting adults in their second and third months of therapy for new smear positive pulmonary TB, at the same facilities where health system costs were ascertained. To estimate government TB expenditures, we surveyed administrators of 8 rural, and 8 urban health facilities, including 3 TB sanatoria, 5 general hospitals, 5 general clinics, and 3 TB dispensaries. This study was approved by the Institutional Review Board of McGill University, and the National TB control programme of Haiti.

Costs for DOTS implementation and maintenance were based on a DOTS expansion project in Ecuador [48], pro-rated to Haiti, based on their respective per capita gross national incomes [49]. Drug costs were calculated from the model estimates of number of new and retreatment cases, and unit costs from the Global Drug Facility [50]. Patients with active TB were assumed to be 50% disabled (productivity loss) from symptom onset until diagnosis, unable to work while hospitalised, and 50% disabled for the remainder of the first two months of treatment [17-19]. Patients who were not diagnosed or who failed treatment were assumed to have 50% disability throughout their illness. Productivity loss from death in HIV uninfected was estimated as per capita annual income [9] times the number of years remaining in the model, with appropriate discounting [46]. For HIV infected the productivity loss was estimated based on the number of years they would be anticipated to survive after development of active TB based on total survival of 9.8 years (35) and the number of years they had survived with HIV infection before developing active TB.

### Sensitivity analyses

We conducted extensive one-way sensitivity and threshold analyses, to assess the robustness of our findings to variations in key assumptions. Each parameter was varied individually, except for pathogenetic parameters that were based on well characterized, carefully studied cohorts. We examined the effect of varying rates of decline in tuberculosis incidence following DOTS expansion, increasing HIV seroprevalence and drug resistance, case detection rate, and higher costs of DOTS implementation, maintenance and drugs. Where possible, sensitivity analyses reflected ranges from the published literature. If these were not available, other variations were used such as halving or doubling costs. We also modelled composite "best case" and "worst case" scenarios, based on combinations of favourable and unfavourable assumptions for influential model assumptions – reflecting findings of the initial one-way sensitivity analyses.

## Results

As shown in Table 3, current government expenditures averaged $432 per TB patient, of which hospital services accounted for 64%, and TB drugs less than 5%. For the 84 TB patients and families surveyed, total TB related out-of-pocket expenses, and lost income averaged $334 – equivalent to 76% of average per capita income of Haitians in 2002. Of 34 patients who could compare earnings before onset of TB and at the time of the survey, 9 (26%) had a significant drop in income (average 65%), while only one (3%) reported increased income.

With the status quo strategy, 226,590 TB cases, and 107,070 TB-related deaths are projected to occur in Haiti over 20 years (Table 4). This morbidity and mortality will result in total societal costs of $378 million, including government costs of $59 million. The DOTS expansion strategy is projected to avert 63,080 TB cases, prevent 53,120 TB deaths, and result in societal savings of $131 million, over 20 years. Of the societal savings, 73% will result from deaths averted, and 20%, or approximately $26 million would reflect savings for patients and their families.

We project a reduction in government expenditures for hospital services from $38 million to $22 million. However these savings would be offset by the need for initial investment for DOTS expansion, plus increased recurrent government expenditures for smear microscopy, directly observed treatment, multi-drug resistant (MDR) TB treatment, supervision, training, and quality control. As a result, net government savings are projected to be only $4 million over the full 20 years.

The substantial societal savings with DOTS expansion was robust in all sensitivity analyses, including doubling the prevalence of drug resistance, or doubling the costs for initial DOTS expansion, for TB drugs, or for ongoing supervision and training (Table 5). Of note – even if the incidence did not decline at all under DOTS expansion, DOTS is still predicted to result in substantial society savings through reduced mortality and reduced costs for patients and their families. However, government savings were much more susceptible to changes in assumptions- reflecting the very modest government savings in the base case analysis. Haitian government savings would be significant only if foreign donors supported the costs for DOTS expansion.

**Figure 2:**
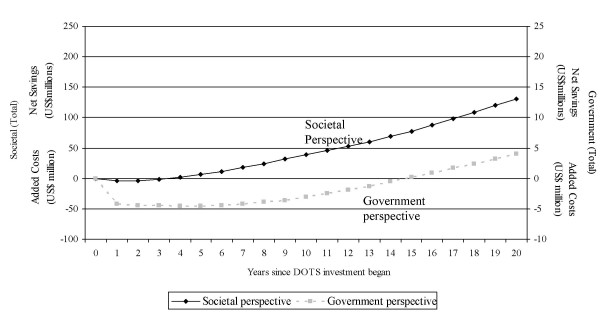
Year by year costs or savings with DOTS expansion in Haiti from societal and government perspectives. *Dashed line *– government perspective, *solid line *– societal perspective.

**Figure 3:**
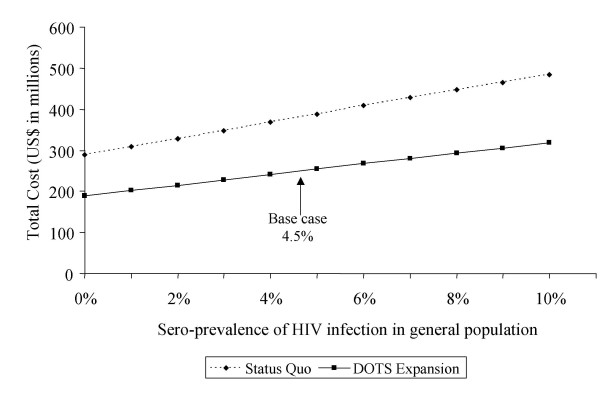
Effect of differences in HIV sero-prevalence on the projected cumulative total TB related costs over 20 years in Haiti with the two strategies. *Dashed line *– status quo, *solid line *– DOTS expansion.

**Figure 4:**
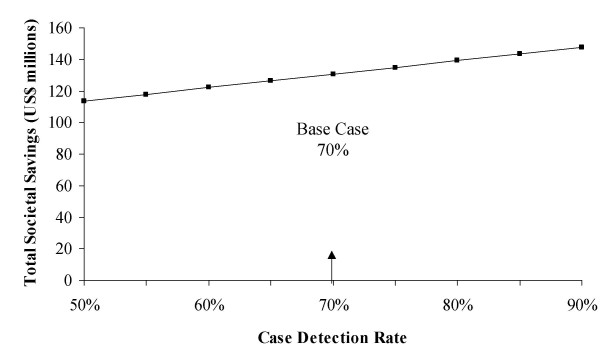
Effect of changes in the case detection rate with DOTS expansion on total societal savings over 20 years in Haiti.

As seen in Figure 2, the DOTS expansion strategy would begin to result in societal savings within 4 years, although government savings would only begin after 15 years. A small increase in HIV sero-prevalence would result in a substantial increase in the number of TB cases, deaths, and TB-related societal costs (Figure 3), although savings with DOTS would be greater. As seen in Figure 4, societal savings would increase by more than $4 million for every 5% increase in case detection. This reflects the lower mortality that would result when more smear positive cases are detected and treated.

## Discussion

We project that DOTS expansion in Haiti, to reach WHO targets, would cost an initial $4.2 million and result in 28% reduction in TB cases, 49% reduction in mortality and net societal savings of $131 million over 20 years. However, we project that the Haitian government would have to make significant initial investment and only begin to achieve savings after 15 years. Taken together, these findings should provide a powerful argument for foreign donors to support initial DOTS expansion in Haiti.

Several findings deserve comment. We have projected savings totalling $26 million for patients and their families from DOTS expansion. Among the patients surveyed, direct out-of-pocket expenditures and lost income represented 49% and 27% respectively of the average *yearly *per capita income in Haiti. And patients surveyed also reported a substantial drop in income at the time of the survey compared to prior to onset of their illness. Savings are anticipated with DOTS expansion, because the decentralisation of diagnostic and treatment services should result in faster diagnosis [17-19], reduced hospitalisation [15,16], and reduced out-of-pocket expenses for treatment and follow-up. Such projected benefits should make DOTS expansion a top priority for donors supporting the Millennium Development goal of poverty reduction [51].

The estimated current government expenditures of $432 per TB patient were surprisingly high – very close to the average per capita income of $440 [9]. We project potential savings from reduced hospitalization, but these will only be realized if hospital expenditures are actually reduced (i.e., by closing beds and reducing staffing). As well any such savings will be offset by increased expenditures for directly observed treatment, and staff supervision, quality control and training. These costly activities are essential for maintenance of a proper DOTS programme [52], but are not necessarily components of TB control programmes prior to DOTS expansion. Therefore new positions must be created, and new workers found with different knowledge and skills – a formidable challenge in many low-income countries. And, immediate government expenditures are required, while savings will only begin after 15 years. As Harold Wilson pointed out "a week is a long time in politics"; most governments will consider 15 years far too long to wait for a payback. Hence, there is little immediate incentive for the Haitian government to implement DOTS.

Another important component of projected government expenditures following DOTS expansion will be lab costs, totalling $9.6 million, or 17% of all TB related government expenditures over 20 years. This reflects the high labour costs for smear microscopy [53], which can take up to 20 minutes of technician time per specimen [52]. In Peru only 2% of TB suspects investigated are smear positive [54], meaning that up to 150 negative smears are examined for each new case found. If treatment success exceeds 85%, enhanced case detection is essential for the long-term epidemiologic impact of DOTS, given the high mortality, and contagiousness of undiagnosed cases. However, given our projections of substantial expenditures for smear microscopy, a high priority should be given to replacing this labour intensive cornerstone of the DOTS strategy.

This study is novel in that we conducted direct surveys to ascertain costs of health facilities, the national TB programme, and TB patients themselves. In this impoverished nation, patient and government expenditures for TB are remarkably high. We combined this direct data gathering with estimates of disability, costs for DOTS expansion, and TB drugs from published experience elsewhere. Additional strengths include the complex decision analysis model which incorporated five TB related health states (with one of three underlying TB drug resistance states) and three HIV related health states within the same model. As well published estimates of risk of TB and HIV infection, HIV progression, development and outcomes of active TB, and mortality were used.

However, there were a number of important potential limitations of this decision analysis. These included our assumptions regarding impact of DOTS on incidence, prevalence of HIV and drug resistance, stability of the population, methods of calculation of annual risk of infection, and costs for DOTS expansion. The assumption that incidence would decline 6% annually following DOTS expansion was based on observations in Peru after nationwide DOTS implementation [5]. This rate of decline is midway between two recent estimates – of 4.3% annually observed with DOTS in China [55], and 7.5% annually predicted for countries achieving WHO targets [56]. And, in sensitivity analyses, societal savings were still substantial even with the extreme assumption of no change in incidence at all. We calculated annual risk of new TB infection from estimated incidence using the Styblo formula [14] – which has been criticized [57]. However, the same formula was applied to both strategies in all years, so inaccuracies in the estimate of infection rates would have been similar for both strategies. We may have over-estimated societal savings because we did not account for other illnesses causing health care costs or disability among those who survived with active TB. However these potential costs should be relatively low since the average age of the patients with active TB in Haiti was 34 so that even those with the most gains in survival would only be 54 by the end of 20 years. As well we did account for mortality from HIV infection (from published studies), and all other causes (from WHO life tables for the general population of Haiti). Thus the most important future societal costs among the added survivors with DOTS, were accounted for in the analysis.

Costs for DOTS expansion are difficult to estimate as there is little published experience to date. For this analysis, costs for expansion were based on published experience in Ecuador [48]. Given the social, economic, and epidemiologic differences between Ecuador and Haiti, these estimates may not be considered valid for Haiti. However these estimated costs were higher than actual costs incurred for a DOTS expansion project in India [58] where the economic situation is very similar to Haiti [9].

A very important limitation of our analysis is the assumption that basic government health services would continue to operate. Therefore we only accounted for the additional costs of DOTS expansion and maintenance. Continued basic government health services in Haiti, depends upon political stability, which is among the worst in the Americas. This problem has contributed to the current problems of the national TB control programme. But, the DOTS strategy has been successfully implemented and maintained amidst major civil conflicts in Mozambique and Nicaragua. Therefore we believe our findings should not be dismissed as overly optimistic by foreign donors considering investment in TB control in Haiti.

We assumed that HIV seroprevalence would remain constant over the next 20 years even though HIV seroprevalence has risen in most developing countries over the past two decades. However if HIV seroprevalence did increase, with a corresponding increased incidence of TB, societal saving would be greater with DOTS (Figure 3), because of improved case detection with corresponding reduction in mortality of undiagnosed active TB in HIV infected. We did not model the potential effect of large scale provision of antiretroviral therapy (ART). This is just being introduced in Haiti and other low income countries, so the costs, efficacy, and population impact of ART are currently unknown. Our assumption of unchanged drug resistance may be incorrect. However, in sensitivity analysis, even when the prevalence of drug resistance was doubled, results were very similar. We also assumed no population growth for Haiti, despite current annual growth of 1.4% [23]. However, a larger population would simply mean more new infections, and new active cases – with either strategy. And, as with higher HIV sero-prevalence, DOTS would be even more cost-saving relative to the status quo strategy.

We have projected that DOTS expansion in Haiti could prevent a large number of TB cases, and TB deaths, with substantial resultant societal savings. But this would require significant Haitian government investment – which may be difficult to ensure, given current political instability and the prospect of little payback after many years. Given this, and the substantial potential humanitarian, economic, and public health benefits, we conclude that foreign donors should strongly consider investing in DOTS expansion in Haiti.

## Competing interests

The author(s) declare that they have no competing interests.

## Authors' contributions

*V. J*. – Conception of project, data gathering, drafting manuscript

*W. M*. – Data gathering and critical revisions of manuscript

*K. S*. – Conception of project, data analysis, critical revisions of manuscript

*O. O*. – Conception of project, data gathering, data analysis, critical revisions of manuscript

*G. B*. – Conception of project, data analysis, critical revisions of manuscript

*F. G*. – Conception of project, and critical revision of manuscript

*D. M*. – Conception of project, data gathering, data analysis, drafting manuscript

## Pre-publication history

The pre-publication history for this paper can be accessed here:



## Figures and Tables

**Figure 1:**
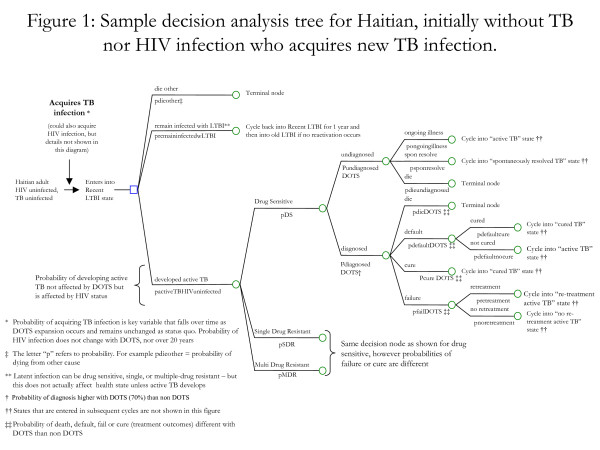
Sample decision analysis tree for Haitian adult, initially without TB infection, nor HIV infection who acquires new TB infection.

**Table 1:** Summary of key input data for Haiti in 2002(All costs in US dollars)

**EPIDEMIOLOGIC/PROGRAMME DATA**	**HAITI**	**REFERENCE/SOURCE**	
Population (2002)	8.3 million	[9]	
Gross National Income annual per capita (US$)	$ 440	[9]	
Life expectancy at birth	52.0	[9]	
All cause mortality	Age specific	[44]	
Incidence new smear positive TB per 100,000 (2001)	138	[8]	
Annual risk of TB infection (ARI)	2.26%	Calculated from [14] and [8]	
Prevalence of LTBI at age 20	41%	Calculated from [14]	
Likelihood of diagnosis and treatment of LTBI	1%	[59] *	
Completion of LTBI Treatment	67%	[60]	
Efficacy of 9 INH – INH Sensitive	90%	[61]	
INH Resistant	0	[62]	
Prevalence of HIV infection – 2002	4.5%	[10]	
Incidence of HIV infection	0.46%/year	Calculated from [35]	
DOTS Coverage – 2002	37%	[11]	
Case detection rate – 2001	49%	[8]	
Drug Resistance			
Single drug resistance	20% (11%-31%)	[12]	
Multi drug resistance (HR)	0.3% (0–4%)	[12;63]	
Treatment outcomes New cases – 2001	Overall [8]	DOTS areas (NTP)	Non-DOTS
Cure/complete	71%	85%	63%
Default/transfer/not evaluated	23%	9%	31%
Die	5%	5%	5%
Fail	1%	1%	1%
Outcomes Re-treatment cases – 2001	Overall [8]	DOTS areas (NTP)	Non-DOTS
Cure/complete	54%	80%	39%
Default/transfer/not evaluated	29%	4%	44%
Die	8%	8%	8%
Fail	8%	8%	8%

Notes:

* – This assumes only dually infected (HIV and TB infection) whose HIV infection is detected and are tuberculin tested at a limited number of screening centres will be treated.

NTP – data on treatment outcomes in areas with DOTS, from cohort reports to National TB programme

**Table 2:** Probabilities of outcomes with different TB and HIV health states

**PATHOGENETIC FACTOR**	**BASE**	**RANGE**	**REFERENCE**
Reactivation from latent TB infection			
Present more than 2 years ("long-standing LTBI")*			
HIV uninfected	0.1%/year	0.1% – 0.2%/year	[28;29]
HIV infected – asymptomatic	3.4%/year	3.4% – 8.7%	[36;64;65]
HIV infected – AIDS	33%/year	33% – 67%	[36]
Within 2 years of new TB infection ("recent LTBI")			
HIV uninfected	5%	2% – 15%	[24;66]
HIV infected – asymptomatic	33%	33% – 100%	Extrapolated
HIV infected – AIDS	100%	50% – 100%	[42;43;67–69]
Within 2 years following re-infection			
HIV Uninfected	1%		[27;70]*
HIV infected	33% or 100%		Assumption
Outcomes of untreated smear positive TB			
Mortality – 1 year, & 2 years	33%, & 50%		From [71]
Spontaneous remission	25%		[72]
Relapse after spontaneous remission	2.5%/year	1.3% – 2.5%/year	[72;73]
Outcomes of treated smear positive TB			
Relapse after cure (total over next 2 years)	3.0%	1.5% – 5%	[74–78]
Cure rate if default (SDR or drug sensitive) **	62.4%		[31–34]
Effect of drug sensitivity or treatment outcomes			
Relative risk of failure/if single drug resistant	2.0		[79]
Relative risk of failure/if multi-drug resistant	10.5		[79]
Relative risk of death/if single drug resistant	1.0		[79]
Relative risk of death/if multi-drug resistant	4.5		[79]
If MDR – Probability of cure with treatment	48%	48%-73%	[22;80]
- Probability of death with treatment	12%	12%-26%	[22;80]
HIV Infected and TB			
Average duration of HIV infection – Total	9.8 years	7.3–9.8	[35;81]
- Time spent in HIV asymptomatic state	9.0 years		[35]
Annual risk of progression of asymptomatic HIV to AIDS	7%	7%-9%	[35;81]
Annual risk of death from HIV: HIV asymptomatic state	4.6%		[35]
Annual risk of death from HIV: AIDS	22%		[35]
Effect of prior active TB on relative risk of death from HIV	2.2	(2.2 – 4.0)	[3;40;41]
Effect of HIV infection on relative risk of death during TB treatment (drug sensitive or single drug resistance)	2.25		[37–39;82]
Relapse after successful TB treatment (cured)	3.1%	3.1% – 6.4%	[83–85]

* Assume that rate of reactivation more than two years after TB infection is the same whether it is after a first infection, or after re-infection.

** Transfer out considered equivalent to default [30]. Overall cure rate if default based on timing of default (from [31]), and cure rates from trials of very short course treatment [32–34].

**Table 3:** Summary of health system and patient costs in Haiti

	**MEAN**	**(SD)**	**SOURCE**
Pre-Diagnosis			

Total Time (onset of symptoms to diagnosis)	4.4 months	(3.5 months)	PCQ
Number of Visits	4.7	(7.2)	PCQ
Cost to health system for visits (total)	$16.31		
Lab costs (per patient – 3AFB smears)	$4.46	(--)	[53]
Patients out-of-pocket: for visits (total)	$40.55	($138)	PCQ
Miscellaneous	$22.87	($116.85)	PCQ
Lost income for patient/family: for visits	$6.19*		PCQ
Miscellaneous	$61.71*		PCQ

Hospitalization			

N (%) hospitalized	47 (56%)		
Average length of stay (for all 84)	21.3 days	(27.2)	PCQ
Health system costs (per patient)	$321.00	(--)	HFQ
Patient out-of-pocket (per hospitalization)	$92.69	($323.41)	PCQ
Lost income for patients and family	$29.40*	($44.37)	PCQ

Direct Observation of Treatment (DOT)			

Number of visits	75	--	NTP
N (%) on DOT	43 (51%)	--	
Health system costs: for DOT (total)	$48.75	--	HFQ
For drug costs (new case)	$20.93	--	**
Patient out-of-pocket expenses (total)	$56.25	($141.00)	PCQ
Lost income for patient and family	$20.40*	($25.50)	PCQ

Follow-up (Medical Check Up)			

Number of visits	6	--	NTP
Health system costs (total)	$20.82	--	HFQ
Patient out-of-pocket expenses	$1.88	($11.65)	PCQ
Lost income for patient and family	$2.35*	($3.67)	PCQ

Total cost per TB patient treated			

Health system	$432.27		
Patient and Family: out-of-pocket costs	$214.24		
Lost Income	$120.05		
Total patients and families	$334.29		

Notes:

* Income = $0.17 (US) per hour based on average per capita GNI ($440) (reference 9)/2496 hours (= 48 hours × 52 weeks)

PCQ = Patient cost questionnaire

HFQ = Health facility questionnaire

NTP = National TB programme guidelines

** = Prices for drugs in DOTS areas from [86], and 1.4 times higher in non-DOTS areas [15;16]

**Table 4:** Projected cumulative TB incidence, related mortality, and costs with two strategies for TB control in Haiti: base case analysis

			**TOTAL COSTS (SAVINGS) ($US MILLIONS)**	
			
			**PERSPECTIVE**	
	**TOTAL TB CASES**	**TOTAL TB DEATHS**	**SOCIETAL**	**GOVERNMENT**

**AFTER ONLY 5 YEARS**				
- Status Quo	64,740	25,730	$77	$16
- DOTS expansion	59,760	19,090	$70	$21
- Cases or deaths averted and added costs or (net savings) with DOTS	4,980	6,640	($7)	$5
**AFTER 10 YEARS**				
- Status Quo	125,330	54,780	$173	$32
- DOTS expansion	103,750	34,030	$134	$35
- Cases or deaths averted and added costs or (net savings) with DOTS	21,580	20,750	($39)	$3
**Over Full 20 Years**				
- Status Quo	226,590	107,070	$378	$59
- DOTS expansion	163,510	53,950	$248	$55
- Cases or deaths averted and (net savings) with DOTS	63,080	53,120	($131)	($4)

**Table 5:** Sensitivity analysis for projected savings over 20 years with DOTS expansion in Haiti

			SAVINGS in $US MILLIONS WITH DOTS EXPANSION *	
			
			**PERSPECTIVE**	
**PARAMETER VARIED**	**CHANGE**	**NEW VALUE**	**SOCIETAL**	**GOVERNMENT**

Base Case (from Table 4)	Base	Base	($131)	($4)
Initial DOTS investment	Double	$8.4 million total	($126)	($0)
Annual DOTS maintenance	Double	$722,000 per year	($128)	($1)
Annual DOTS maintenance + cost of TB drugs	Double both	$847,900 per year	($128)	($1)
Foreign Donor Pays: Initial + annual + TB drugs	Eliminate all three	$8.44 million + $847,000 per year	($138)	($11)
Change average duration of hospitalization	Decrease with DOTS, increase with Non-DOTS	3 days – DOTS 60 days non-DOTS	($165)	($29)
Single (SDR) and Multi (MDR) Drug resistance	Double prevalence of drug resistance	SDR : 40% MDR : 0.6%	($131)	($4)
Impact of DOTS expansion on TB incidence in subsequent years	None 1/3 of base 2/3 of base	0% annual decline 2% annual decline 4% annual decline	($61) ($88) ($111)	$ 14 * $ 7* $ 1*

Notes:

* A number in parentheses indicates net societal or government savings, while a number not in parentheses indicates a net increased cost with DOTS expansion,.
